# COVID-19 Disease in Syrian Patients With Cancer: Clinical Manifestations, Laboratory Findings, Treatment, and Outcomes

**DOI:** 10.1200/GO.21.00283

**Published:** 2022-03-01

**Authors:** Michel Najjar, Sara Albuaini, Mohammad Fadel, Ahmed Aljbawi, Fatema Mohsen, Seham Sulaiman, Abir Koudsi

**Affiliations:** ^1^ Faculty of Medicine, Syrian Private University, Damascus, Syria; ^2^ Department of Internal Medicine, Faculty of Medicine, Damascus University, Damascus, Syria; ^3^ Department of Internal Medicine, Faculty of Medicine, Syrian Private University, Damascus, Syria; ^4^ Department of Family and Community Medicine, Faculty of Medicine, Damascus University, Damascus, Syria; ^5^ Department of Family and Community Medicine, Faculty of Medicine, Syrian Private University, Damascus, Syria

## Abstract

This study aims to describe the clinical manifestations, laboratory findings, treatment, and outcomes of patients with cancer with COVID-19 infection in Syria. The primary objective was to identify the overall survival (OS) time, and the secondary objectives were to identify factors associated with severe COVID-19 infection.

**METHODS:**

This multicenter retrospective study was undertaken at four hospitals in Damascus, Syria, between March 28, 2020, and March 29, 2021. Data extracted from medical records included clinical manifestations, radiologic findings, laboratory results, treatment, and outcomes. Survival analysis was done by using the Kaplan-Meier method and Cox regression model for follow-up and anticancer treatment patients to study the effect on OS time. The effects of potential risk factors of developing severe COVID-19 were studied by multivariable logistic regression.

**RESULTS:**

Of 114 patients included, 61 (53.51%) were male. Smokers represented 29 (25.44%), and 63 (55.26%) patients had a history of coexisting chronic diseases. The most common cancer type was breast cancer 17 (14.91%). Sixty-eight (59.65%) patients were receiving anticancer treatment within 1 month of being diagnosed with COVID-19 infection and 46 (40.35%) were outpatient follow-ups. Multiple logistic regression analysis showed that comorbidities (odds ratio: 2.814, *P* = .044) and anticancer treatment (odds ratio: 8.790, *P* < .05) were risk factors linked to severe to critical COVID-19 infection. OS time was 245 (95% CI, 217.96 to 272.47) days, lower among patients with cancer with COVID-19 infection receiving anticancer treatment compared with follow-up patients (*P* value < .05).

**CONCLUSION:**

Patients with cancer with COVID-19 infection receiving anticancer treatment had a lower OS time. It may be worth considering stopping anticancer treatment in patients with cancer with COVID-19 when possible in search of better outcomes.

## INTRODUCTION

After the first known case of COVID-19, reported in China, December 2019, the virus rapidly evolved into a pandemic, which affected all aspects of life. Although the true number of cases is unknown because of asymptomatic carriers and low testing rates in low-income countries, the latest figures reported more than 186,000,000 confirmed COVID-19 cases and more than 4,000,000 confirmed deaths.^1^ The figures reported in Syria at the time were 25,619 confirmed cases and 1898 deaths.^2^ The severity of COVID-19 infection varies according to the health status of each patient. Studies have shown that older age and the presence of comorbidities can be associated with more severe cases and clinical complications.^3^

**CONTEXT**

**Key Objective**
What factors are linked with severe COVID-19 disease among patients with cancer?
**Knowledge Generated**
Our study showed that the presence of comorbidities, receiving anticancer treatment within 1 month of COVID-19 diagnosis, and receiving corticosteroids to treat COVID-19 were associated with severe to critical disease in patients with cancer. Being on active anticancer treatment is also associated with higher mortality in patients with COVID-19 with cancer. Our study revealed that lymphocytopenia, neutrophilia, low platelets, low hemoglobin, and high levels of AST, ALT, blood urea, C-reactive protein, and D-dimer were laboratory indicators for severe infection.
**Relevance**
Oncologists should consider withdrawing anticancer treatment among patients with cancer with COVID-19 infection when possible in search of better outcomes. Randomized controlled trials should be conducted on patients with cancer with COVID-19 to assess the effect of corticosteroid use among these patients.

Patients with cancer are more vulnerable to COVID-19 infection compared with other individuals; additionally, they experience more serious and severe diseases,^3^ which may be a consequence of an exhausted immune system caused by anticancer therapy and cancer itself. The higher hospital admission rate among patients with cancer because of treatment requirements or cancer complications puts them at increased risk of COVID-19 infection, especially in the lack of serious preventative majors to prevent the repeated outbreaks of COVID-19. As of yet, there is no evidence of the efficacy of vaccines among patients with cancer. Vaccines may not be as effective among the immunocompromised compared with those who have a healthy immune system. Added to the above, the negative effects cancer treatments have on the immune system weaken the potency of the vaccine.^4^ All the above reasons shed concern in the medical community about whether patients with cancer are more at risk of COVID-19–related complications. Also, there is concern regarding the effect of chemotherapy on the ability of patients to fight this virus. To fill these gaps of knowledge, this study was conducted to describe the clinical manifestations, laboratory findings, treatment, and outcomes of patients with cancer with COVID-19 infection in Syria. The primary objective was to identify the overall survival (OS) time and the secondary objectives were to determine the factors linked with the increased risk of severe COVID-19 infection.

## METHODS

### Study Design, Setting, and Participants

This retrospective, multicenter, observational study was conducted at four main Hospitals in Damascus and Rural-Damascus (Damascus Hospital Al-Mujtahid, Ibn Al-Nafees Hospital, Al-Mouwasat Hospital, and Al-Bairoune University Hospital) between March 23, 2020, and March 24, 2021. Damascus Hospital and Ibn Al-Nafees Hospital are affiliated with the Syrian Ministry of health, whereas Al-Mouwasat Hospital and Al-Bairoune University Hospital are affiliated with the Syrian Ministry of Higher education and Scientific Research. It is important to know that Damascus Hospital and Al-Mouwasat Hospital were two emergency hospitals involved in the isolation and management of patients with COVID-19 during the outbreak. After reviewing 2,304 COVID-19 medical records, a total of 114 patients with cancer with confirmed COVID-19 diagnoses were enrolled in this study.

#### Inclusion criteria.

Inclusion criteria were laboratory-confirmed COVID-19 infection according to the WHO published criteria^5,6^ and history of confirmed malignancy.

#### Exclusion criteria.

Exclusion criteria were suspected and probable cases that were not confirmed with positive reverse transcription-polymerase chain reaction (RT-PCR) assay; inadequate data regarding cancer treatment and patient's health status before COVID-19 diagnosis; and patients who were discharged from the hospital against medical advice, and therefore missed outpatient follow-up.

Patients were classified into two groups: (1) patients on active cancer treatment (including chemotherapy, surgery, radiotherapy, immunotherapy, targeted therapy, and hormonal therapy) within 1 month at the time of COVID-19 diagnosis, and (2) follow-up patients (defined as patients who were not taking any of the above-mentioned treatments > 1 month at the time of diagnosis with COVID-19).

The authors collected clinical data from medical records and contacted patients via telephone where data from files were incomplete. Data included sociodemographic features, clinical presentations, laboratory results on admission, radiologic assessments, recent exposure history, the result of RT-PCR, COVID-19 treatment, and clinical outcomes. This study was approved by the Ethics committee of Damascus Hospitals of Syria Ministry of Health. Two investigators separately checked the data collection to confirm the accuracy of the data gathered.

### Study Definitions

Coronavirus infection was confirmed according to WHO published criteria with positive RT-PCR assay of nasal and/or pharyngeal specimen. Patients were classified into two groups: mild to moderate and severe to critical patients on the basis of the National Institutes of Health's COVID-19 treatment guidelines.^7^

Anemia was defined as hemoglobin < 13 g/dl (130 g/L) in men and hemoglobin < 12 g/dl (120 g/L) in women. Neutrophilia was defined as a neutrophil count of more than 7.5 × 10^9^/L. Lymphocytopenia was defined as a lymphocyte count of < 1,000 × 10^9^/L.

Description of computed tomography (CT) and chest x-ray including abnormal findings was reviewed by an attending physician in the Respiratory Department.

Acute respiratory distress syndrome (ARDS) was diagnosed on the basis of the guidance of WHO for COVID-19.^8^

### Statistical Analysis

Continuous variables were expressed as the mean with standard deviation or as a median with an interquartile range as appropriate (descriptive statistics). Categorical variables were represented as frequencies percentages. The *t*-test and chi-square had been used as appropriate to compare between COVID-19 severity (mild to moderate and severe to critical patients) laboratory findings, and antibiotic therapy. OS was defined as the time from the onset of COVID-19 symptoms to death from any cause or survival till the last follow-up (March 24, 2021). Survival analysis was done by using the Kaplan-Meier method and compared using the log-rank test. Survival analysis was done using the Cox regression model performed with the Wald test for follow-up and anticancer treatment patients to study the effect on their survival time. The effects of potential risk factors of developing severe COVID-19 were studied by multivariable logistic regression. The odds ratio (OR) and corresponding 95% CI were calculated using multivariable logistic regression. All statistical analyses were performed using Statistical Package for Social Sciences (SPSS) statistics version 23.0. *P* values < .05 were considered statistically significant.

## RESULTS

### Sociodemographics and Clinical Characteristics of Patients With Cancer With COVID-19

After reviewing the medical records of 2,304 patients with COVID-19, a total of 114 (4.95%) patients with a previous diagnosis of cancer met the inclusion criteria of this study. The COVID-19 infection rate among patients with cancer was 4.95% (one case per 20 people). The median age was 58 years, and the range was between 15 and 90 years. Males represented 61 (53.51%) and females represented 53 (46.49%). Fifty-nine (51.75%) patients had severe to critical COVID-19 infection, whereas 55 (48.25%) of patients had mild to moderate infection. The median (interquartile range) of the body mass index (BMI) was 26.27 (22.18-30.08). The median BMI was found to be higher among severe to critical patients 26.99 (21.84-31.79) than those with mild to moderate infection 25.43 (22.58-27.34). Twenty-nine (25.44%) patients were current or previous smokers; among those, 19 (32.20%) patients developed severe to critical COVID-19, whereas 10 (18.18%) patients had mild to moderate infection (Table 1).

**TABLE 1:**
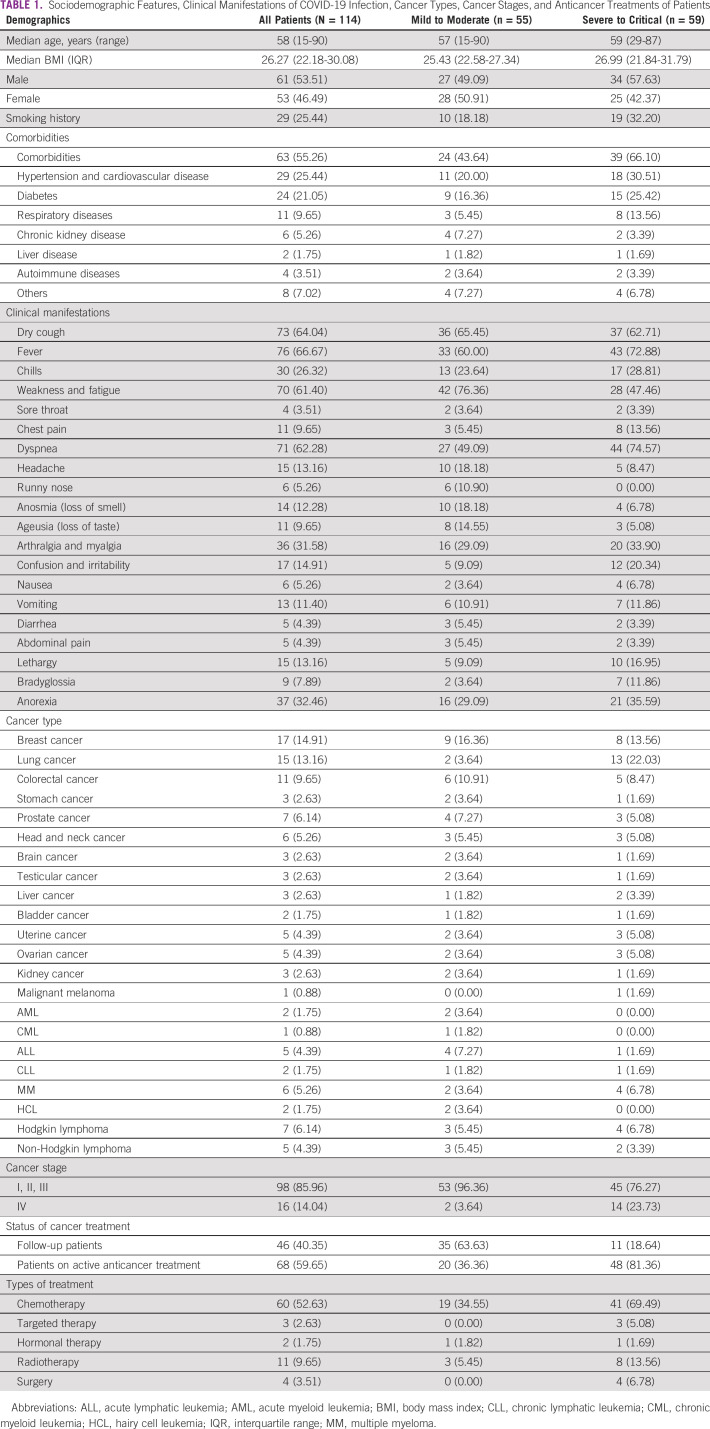
Sociodemographic Features, Clinical Manifestations of COVID-19 Infection, Cancer Types, Cancer Stages, and Anticancer Treatments of Patients

Sixty-three (55.44%) patients had one or more coexisting chronic disease(s). The most common comorbidities were hypertension and cardiovascular disease 29 (25.44%), followed by diabetes 24 (21.05%) and respiratory disease 11 (9.65%; Table 1).

The predominant clinical presentation among severe to critical COVID-19 cases were dyspnea (n = 44; 74.57%), fever (n = 43; 72.88%), and dry cough (n = 37; 62.71%), whereas weakness and fatigue (n = 42; 76.36%) was the most common clinical symptom among patients with mild to moderate COVID-19 cases (Table 1).

### Cancer Types, Stages, and Treatment

**FIG 1:**
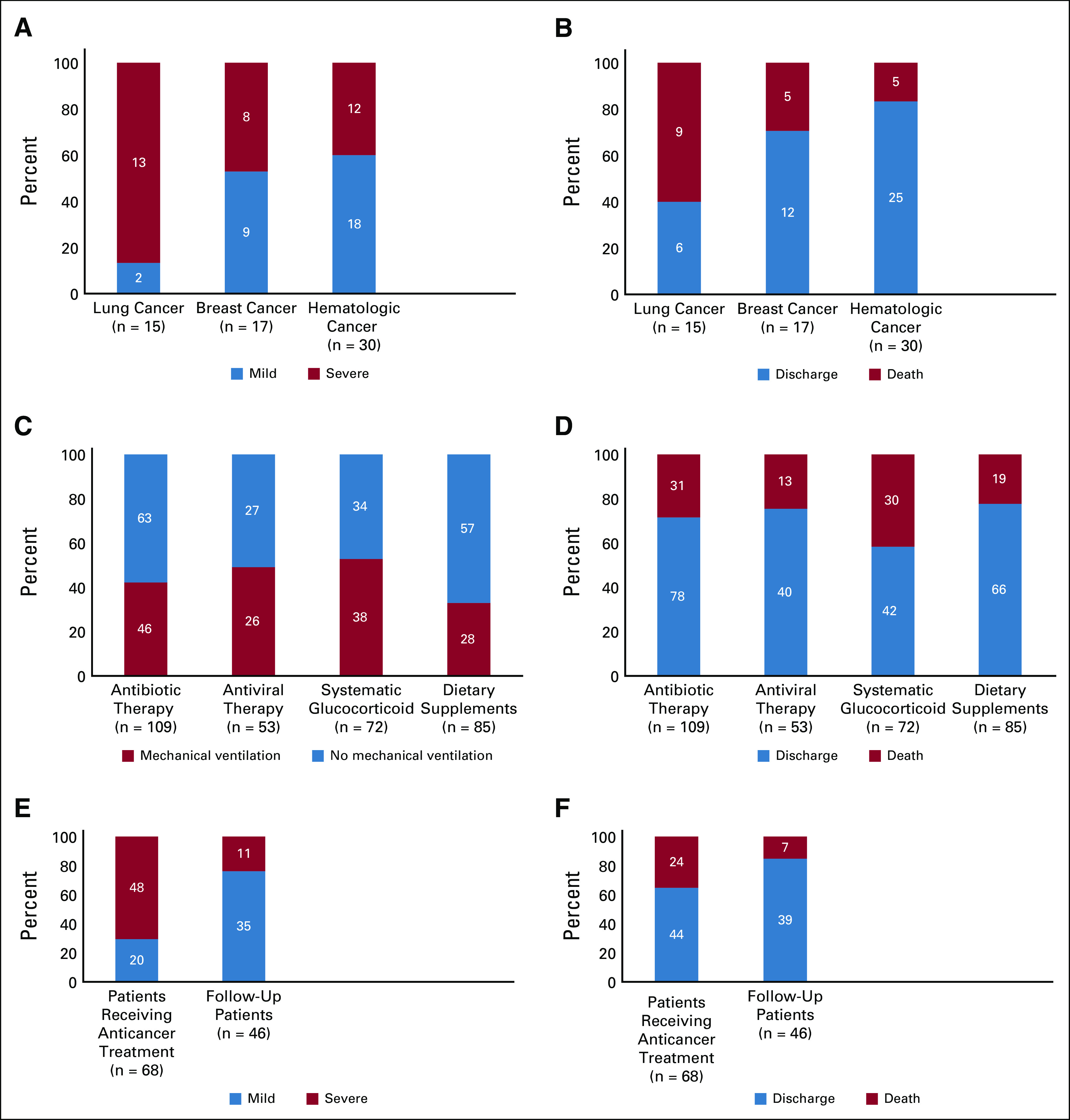
Comparison of COVID-19 severity and mortality for different cancer types and cancer treatment status. (A and B) Comparison between patients with breast cancer, lung cancer, and hematologic cancer. (C and D) Comparison between patients on active anticancer treatment and follow-up patients. (E and F) Comparison of mortality and mechanical ventilation requirements between different COVID-19 treatment options (antibiotics, antiviral, systematic glucocorticoid, and dietary supplements).

The most common cancer type was breast cancer (n = 17; 14.91%), lung cancer (n = 15; 13.16%), and colorectal cancer (n = 11; 9.65%). Cancer staging was done using the American Cancer Society criteria.^9^ Sixteen (14.04%) patients were in stage IV, whereas 98 (85.96%) were in stage I, II, and III of the disease. Stage IV tumors were dominant in severe to critical COVID-19 infection 14 (23.73%) versus two (3.64%) in mild to moderate cases. Sixty-eight (59.65%) patients received anticancer treatment within 1 month before COVID-19 diagnosis, 60 (52.63%) of them received chemotherapy, three (2.63%) received targeted treatment, 11 (9.65%) received radiotherapy, two (1.75%) received hormonal therapy, and only four (3.51%) had surgical treatment. Forty-six (40.35%) patients were follow-up patients (Table 1). Among the 11 patients who received radiotherapy treatment, four patients had breast cancer, two of whom were additionally receiving chemotherapy and hormonal therapy; three patients had lung cancer and were additionally receiving chemotherapy; two patients had head and neck cancer, one of whom had thyroid cancer and was additionally receiving chemotherapy, and the other had larynx cancer and was additionally receiving chemotherapy; one patient had brain cancer and was additionally receiving chemotherapy; and one patient had liver cancer and was additionally receiving chemotherapy. Patients with lung cancer had more severe disease and a higher mortality rate compared with breast and hematologic cancer (Figs 1A and 1B).

### Laboratory and Radiologic Findings

The median time from the onset of symptoms to hospital admission was 6 days (range between 1 and 20 days). Chest CT was requested for 106 (93%) patients on admission. The most common chest CT findings were bilateral peripheral patchy consolidation (n = 69; 65.09%) and ground-glass opacity (n = 58; 54.72%).

The neutrophil count was significantly higher among the severe to critical group (8.24 × 10^9^/L) compared with the mild to moderate group (5.68 × 10^9^/L; *P* value < .05). Both mild to moderate and severe to critical COVID-19 infection groups had anemia 10.81 g/dl and 9.67 g/dl, respectively (*P* value < .05). Higher levels of C-reactive protein (CRP) and D-dimer were found within severe to critical COVID-19 infection group (73.98 mg/L and 3.57 mg/L) compared with the mild to moderate group (55.53 mg/L and 1.34 mg/L, respectively; *P* value < .05). Lymphocytopenia, neutrophilia, low platelets, low hemoglobin, and high levels of AST, ALT, blood urea, CRP, and D-dimer were linked to severe to critical COVID-19 group (*P* value < .05; Table 2).

**TABLE 2:**
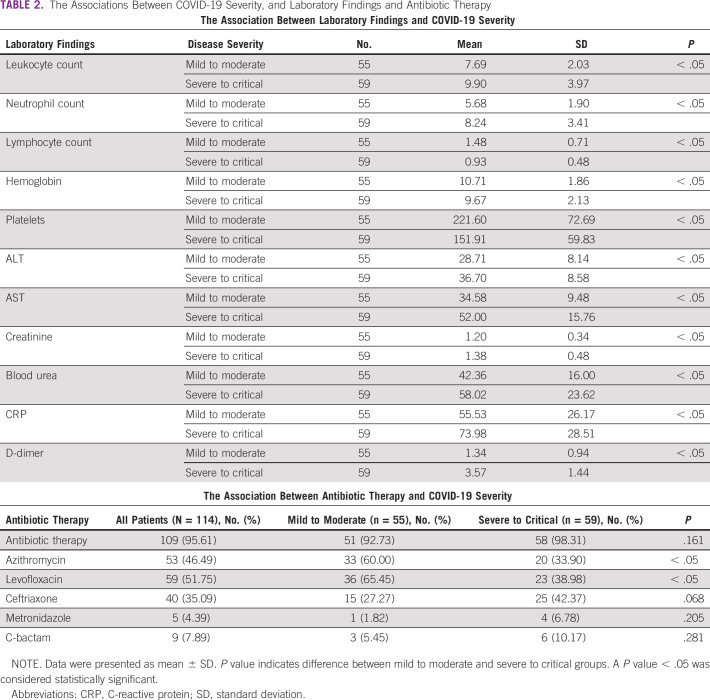
The Associations Between COVID-19 Severity, and Laboratory Findings and Antibiotic Therapy

### Treatment, Complications, and Outcomes

The average hospital stay was 5 days (range between 1 and 35 days). Anticancer treatment was stopped for most patients after confirmation of COVID-19 infection. Patients who were discharged from the hospital were quarantined for 14 days.

Antibiotic therapy was administered to 109 (95.61%) patients. Levofloxacin was the most used antibiotic (n = 59; 51.75%) followed by azithromycin (n = 53; 46.49%). The antiviral therapy was administered to 53 (46.49%) patients, hydroxychloroquine 34 (29.82%) patients, and oseltamivir 26 (22.81%) patients. Systemic glucocorticoid therapy was administered to 73 (63.16%) patients. The dietary supplements (zinc, vitamin C, and vitamin D) were given to 85 (46.49%) patients. A total of 99 (86.84%) required oxygen support, and 46 (40.35%) patients were placed on mechanical ventilation. Complications (n = 51; 44.74%) reported include ARDS (n = 29; 25.44%), myocardial infarction (n = 18; 15.79%), and deaths (n = 31; 27.19%; Table 3). At the time of the last follow-up (March 24, 2021), 31 patients had died: 24 on anticancer treatment and seven follow-up patients. Among the 31 patients, 26 (83.87%) patients died as a direct consequence of COVID-19 infection, whereas five (16.13%) patients died after they had been discharged from the hospital, because of other causes.

**TABLE 3:**
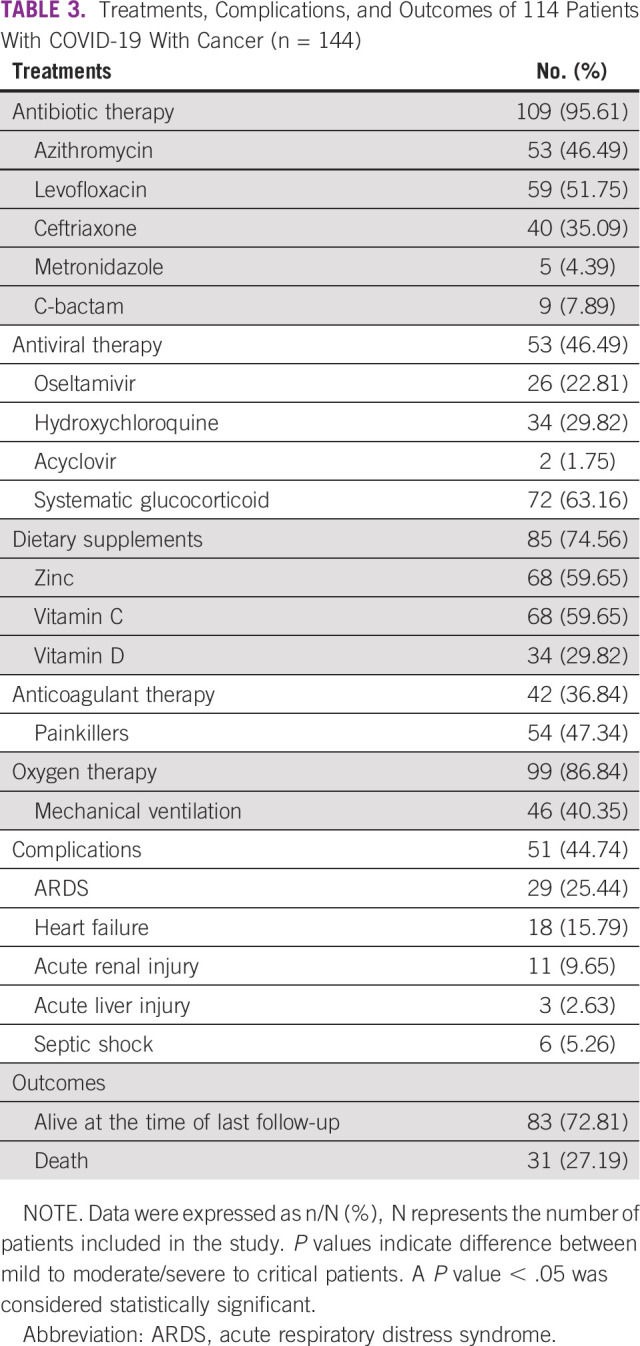
Treatments, Complications, and Outcomes of 114 Patients With COVID-19 With Cancer (n = 144)

Patients receiving systemic glucocorticoids were associated with higher requirements of mechanical ventilation (n = 38; 52.78%) and higher mortality numbers (n = 30; 41.67%) compared with other methods of treatments (*P* < .05; Figs 1C and 1D; Appendix Table A1). Azithromycin and levofloxacin use was significantly linked to mild to moderate disease compared with severe to critical patients (*P* value < .05; Table 2).

### Multivariate Logistic Regression Analysis

Multiple logistic regression analysis showed that comorbidities (*v* no; OR: 2.814, *P* = .044) and anticancer treatment (*v* follow-up; OR: 8.790, *P* < .05) were risk factors linked to severe to critical COVID-19 infection. Other studied risk factors such as age > 60 years (*v* ≤ 60 years; OR: 1.301, *P* = .637), BMI > 30 kg/m^2^ (*v* ≤ 30; OR: 2.837, *P* = .073), male (*v* female; OR: 1.466, *P* = .499), positive smoking history (*v* negative; OR: 1.439, *P* = .542), lung cancer (*v* other types of cancers; OR: 4.980, *P* = .087), and stage IV cancer (*v* I, II, and III; OR: 4.080, *P* = .114) were not linked with severe to critical COVID-19 infection (Appendix Table A2).

### The Effect of Anticancer Treatment on the Clinical Presentation, Disease Severity, and Outcomes

The most common clinical presentation among patients receiving anticancer treatment was fever (n = 49; 72.06%), compared with n = 27 (58.70%) among follow-up patients. Weakness and fatigue were the most frequent symptoms among follow-up patients (n = 36; 78.26%) compared with patients receiving anticancer treatment 34 (n = 34; 50.00%; *P* < .05). Sixty-five (57.02%) patients had a positive contact history with a COVID-19–infected patient, and it was lower among patients on anticancer treatment (n = 35; 51.47%) compared with patients on follow-up (n = 30; 65.22%; Table 4).

**TABLE 4:**
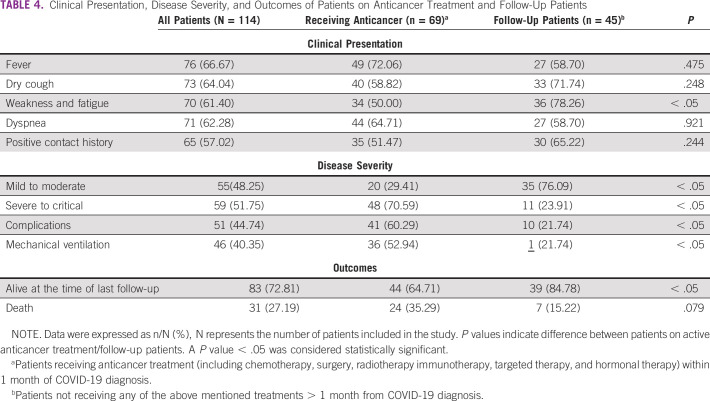
Clinical Presentation, Disease Severity, and Outcomes of Patients on Anticancer Treatment and Follow-Up Patients

Patients on active anticancer treatment were more vulnerable to developing severe to critical COVID-19 infection and poor outcomes including severe complications and the need for mechanical ventilation in comparison with follow-up patients (*P* value < .05; Table 4; Figs 1E and 1F).

**FIG 2:**
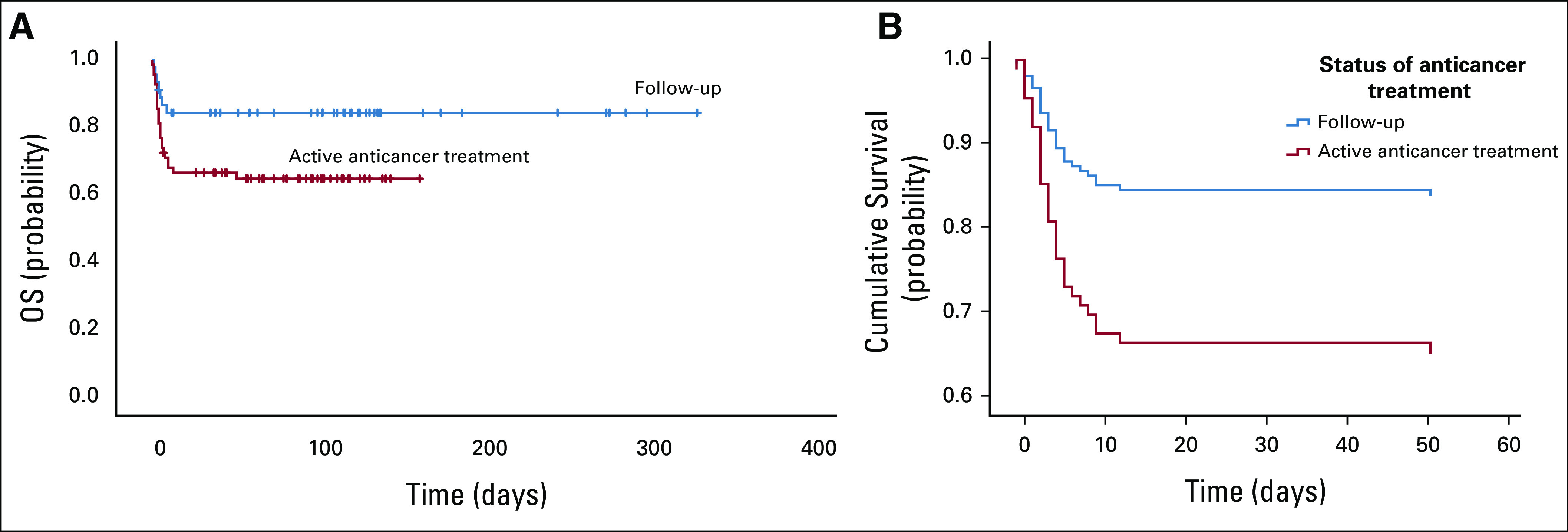
(A) Kaplan-Meier plot of OS of 114 patients with cancer with COVID-19 infection who received anticancer treatment and who were on follow-up. (B) Cox regression survival analysis of the follow-up and anticancer treatment patients, who were diagnosed with COVID-19 infection. OS, overall survival.

The OS time was 245 (95% CI, 217.96 to 272.47) days, and there is a statistically significant difference in OS time between the two groups in favor of the follow-up patient group (*P* value < .05; Fig 2). The mean duration from the onset of symptoms to death or survival was 284 (95% CI, 248.37 to 319.34) days among follow-up patients and 109 (95% CI, 9.17 to 127.38) days among patients on active anticancer treatment (Fig 2A; Appendix Tables A3 and A4). Cox regression test for survival analysis among the two groups was performed, and a statistical difference between the patients on active anticancer treatment and follow-up patients was found (*P* value < .05; Fig 2B; Appendix Table A5).

## DISCUSSION

In this study, we reviewed the impact of COVID-19 infection among 114 patients with cancer. The COVID-19 infection rate among patients with cancer was 4.95% (one case per 20 people), higher than the rate among the Syrian population (0.14%; one case per 701 people).^2^ Syria restricting COVID-19 tests to severe cases accompanied with low testing among the population in Syria^10,11^ may have led to a higher diagnosis of cases among patients with cancer. The majority of patients had a positive contact history with an infected individual, lower among patients with anticancer treatment compared with patients on follow-up. This can be attributed to the frequent hospital admissions patients on anticancer treatment have compared with follow-up patients. This is why we need to implement more strict preventive measures to avoid the spreading of COVID-19 in this group. A staggering 31 (27.19%) patients died in our study; this rate is higher compared with the general population (7.36%) among noncancer patients.^1^ These findings were similar to other studies on patients with cancer.^3^

Our data showed that patients with cancer with comorbidity were at high risk for developing severe disease, and this finding was similar to other studies from New Jersey^12^ and the US Centers for Disease Control and Prevention.^13^ Our study showed an association between receiving anticancer treatment within one month of COVID-19 infection and disease severity, complications, and the requirement of mechanical ventilation, and lower OS time. Lower OS time was found among patients receiving anticancer treatment, and the retrospective review of the cause of death revealed that most patients died as a direct consequence of COVID-19 infection. This important finding highlights a debate with regards to stopping anticancer treatment for individuals with COVID-19 infection and cancer. This was consistent with many previous studies displayed in systematic review and meta-analysis.^14,15^ However, other studies found no significant association between patients on chemotherapy and severe or critical COVID-19 infection.^16^ Regarding clinical presentation and laboratory findings, fever, dry cough, dyspnea, weakness, and fatigue were the most frequent symptoms, which were coherent to those of patients with COVID-19 without cancer.^17^ Regarding laboratory findings in patients with cancer, abnormal hemoglobin levels and lymphocyte count, neutrophil count, blood urea, creatinine, ALT, AST, CRP, and D-dimer levels were linked to severe to critical COVID-19 group. A study in China also reported high levels of CRP and D-dimer among patients with cancer with severe to critical COVID-19.^18^ Another study in Tehran reported an association between high CRP plasma levels and high neutrophil-to-lymphocyte ratio with severe COVID-19 infection.^19^ These abnormal laboratory findings may be attributed to the overproduction of inflammatory cytokines cytokine storm in severe COVID-19 infection.^20^

The most common cancer was breast cancer, similar to a previous study.^12^ Patients with lung cancer had a higher mortality rate (n = 9; 60.00%), compared with breast cancer (n =5; 29.41%) and hematologic cancers (n =5; 16.67%), and this was consistent with other studies.^16^ This may be caused by poor lung function because of the effects of the tumor and the underlying chemotherapy, which may damage lung interstitial or parenchymal tissue.

Fifty-one (44.74%) patients developed complications because of COVID-19 infection. ARDS was the most common complication (n = 29; 25.44%). ARDS was also the leading cause of death among the general population,^21^ and this is probably because of the high expression of angiotensin-converting enzyme 2 in the alveoli, which makes the lungs the main target for the virus.^22^

Antibiotics, antiviral, systematic glucocorticoids, and dietary supplements were used to treat COVID-19 infections in patients with cancer. One hundred nine (95.61%) patients received antibiotic therapy; this is worrisome as COVID-19 is a viral disease and only a few would develop bacterial coinfection. The high percentage may be due to limited treatments caused by the economic sanctions imposed on Syria. Drugs such as remdesivir, baricitinib, and tocilizumab used to manage COVID-19–infected individuals are not available in Syria.^23^ Furthermore, drugs such as hydroxychloroquine and oseltamivir were given to 53 (46.49%) patients, whereas antibiotics had been administered to most patients. There was a statistically significant association between administration of azithromycin and levofloxacin, and mild to moderate disease. Other studies from Spain showed benefits from the administration of azithromycin.^24^ However, the reported effect of azithromycin is often derived from patients treated with hydroxychloroquine and azithromycin combination versus hydroxychloroquine alone, and studies that assess azithromycin monotherapy reported a wide effect range.^25^ Considering the potential antiviral activity of respiratory fluoroquinolones against SARS-CoV-2, along with their immunomodulatory properties, it may be beneficial as an adjunct treatment in COVID-19.^26^

A statistically significant association between the administration of corticosteroids, and higher requirement of mechanical ventilation, and a higher mortality rate was found. This was an unexpected result, especially with the widespread use of corticosteroids in the management of severe COVID-19 cases. However, reports about using corticosteroid therapy for pneumonia or ARDS had always conflicting results. A study from Saudi Arabia showed that the use of corticosteroid therapy in critically ill patients with Middle East respiratory syndrome (MERS) was associated with delayed MERS-coronavirus ribonucleic acid clearance.^27^ Other studies in patients with SARS coronavirus found that high-dose systemic corticosteroid therapy was associated not only with increased subsequent blood viral loads but also with adverse effects and increased mortality.^28,29^ However, several other studies have found contradictory results.^30,31^ COVID-19 and Cancer Consortium (CCC19) found an association between steroid dexamethasone use and increased mortality among patients with cancer. Among the CCC19 analysis, patients receiving high-dose corticosteroids along with any other COVID-19 treatment were twice more likely to die compared with patients treated with other medications or patients not requiring any treatment.^32^

The main limitation of the study is the retrospective aspect of data collection. Issues with data collection included disorganized storage facilities, illegible handwriting, subjective records, and contacting patients via telephone to complete data collection. These issues could be addressed by handing out an admission questionnaire to patients on entry. In addition, converting from medical records to electronic files, making the data more valid and accurate, and easier to access for future studies would eradicate most of the current weaknesses faced by the authors of the study. A second limitation was that not all patients were considered in this study as some records were lost, and some patients admitted to the hospital withdrew and were transferred to another hospital or private hospital for management. To overcome this problem, a prospective multicenter study on a national level should be conducted to offer generalization.

In conclusion, this retrospective study of patients with cancer with COVID-19 infection was linked to the presence of comorbidities and receiving anticancer treatment within one month of COVID-19 diagnosis. Also, patients who were on anticancer treatment had a lower OS time. It may be worth considering stopping anticancer treatment in patients with cancer with COVID-19 when it is possible in search of better outcomes. Also, we advise on strict precautionary measures in hospitals when dealing with patients undergoing cancer treatment. We also found that lymphocytopenia and high levels of CRP and D-dimer were laboratory indicators for severe infection. Regarding COVID-19 treatments, the administration of levofloxacin and azithromycin was associated with mild to moderate disease. Future randomized controlled trials are needed to confirm this benefit. Patients who received corticosteroid had a poor prognosis; randomized controlled trials should be conducted on patients with cancer with COVID-19 to accurately assess the effect of corticosteroid use among these patients.

## Figures and Tables

**TABLE A1:**
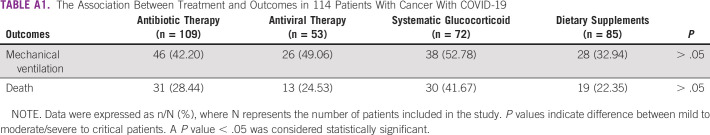
The Association Between Treatment and Outcomes in 114 Patients With Cancer With COVID-19

**TABLE A2:**
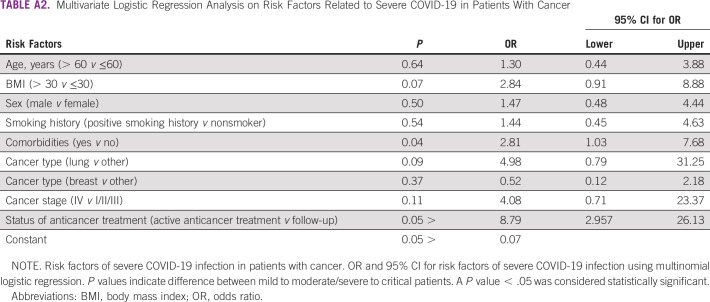
Multivariate Logistic Regression Analysis on Risk Factors Related to Severe COVID-19 in Patients With Cancer

**TABLE A3:**
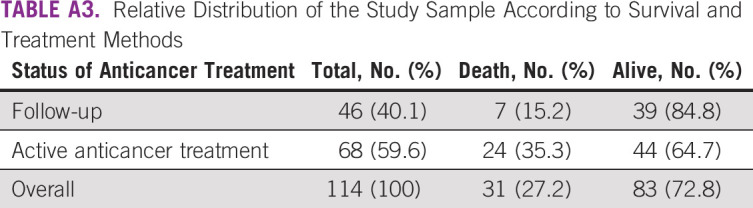
Relative Distribution of the Study Sample According to Survival and Treatment Methods

**TABLE A4:**
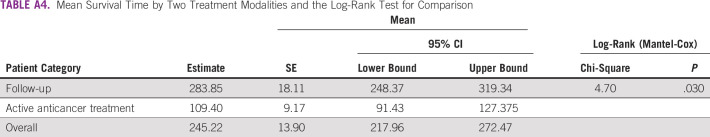
Mean Survival Time by Two Treatment Modalities and the Log-Rank Test for Comparison

**TABLE A5:**
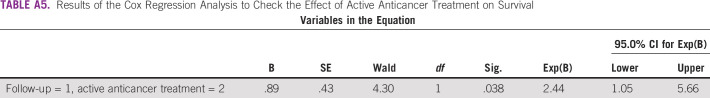
Results of the Cox Regression Analysis to Check the Effect of Active Anticancer Treatment on Survival
